# Composted Agricultural and Forestry Organic Materials Amendment Rates Alter the Fluorescence Characteristics of WE-DOM and Chrysanthemum Growth in a Soil-Based Growing Medium

**DOI:** 10.3390/plants15162541

**Published:** 2026-08-21

**Authors:** Yan Li, Xinyuan Zhang, Yu Hu, Hongsheng Gao, Huawei Yang, Ruixin Bi, Diwei Song, Xiaoxiao Xiong, Dan Wei

**Affiliations:** 1 Heilongjiang Academy of Black Soil Conservation and Utilization, Key Laboratory of Black Soil Protection and Utilization, Heilongjiang Fertilizer Engineering Technology Research Center, Harbin 150086, China; li.yan622@163.com (Y.L.); ghs6837@163.com (H.G.); 2 College of Resources and Environment, Jilin Agricultural University, Changchun 130118, China; zoecy0606@163.com; 3 Institute of Plant Nutrition, Resources and Environment, Beijing Academy of Agricultural and Forestry Sciences, Beijing 100097, China; huyu0805@126.com; 4 School of Resources and Environment, Northeast Agricultural University, Harbin 150030, China; b230201003@neau.edu.cn (H.Y.); b240201011@neau.edu.cn (R.B.); s2602021130@neau.edu.cn (D.S.); 5 College of Life Sciences, Northeast Agricultural University, Harbin 150030, China; 19972771660@163.com

**Keywords:** compost amendment, water-extractable dissolved organic matter, EEM-PARAFAC, fluorescence regional integration, growing media, chrysanthemum, entropy-weighted comprehensive evaluation

## Abstract

To evaluate how composted agricultural and forestry organic materials function as components of horticultural growing media, a pot experiment was conducted with chrysanthemum (*Chrysanthemum morifolium* Ramat.) grown in a cinnamon-soil-based medium. The composted material, produced from chestnut shells, chicken manure, and spent mushroom substrate, was incorporated at 0%, 5%, 10%, 15%, 20%, 25%, 30%, 35%, 40%, 45%, and 50% (*v*/*v*). Water-extractable dissolved organic matter (WE-DOM) was characterized by excitation-emission matrix fluorescence spectroscopy coupled with parallel factor analysis (EEM-PARAFAC), together with the fluorescence index (FI), biological index (BIX), humification index (HIX), and fluorescence regional integration (FRI). Growing-medium physicochemical properties, chrysanthemum traits, and an entropy-weighted comprehensive evaluation were also assessed. Compost amendment increased soil organic matter (SOM), dissolved organic carbon (DOC), total nitrogen, and total phosphorus, lowered pH, and was associated with improved porosity and water-retention characteristics. FI ranged from 1.810 to 2.371 and exceeded 1.9 at amendment rates of 30–35%, suggesting a greater contribution from microbially derived DOM. BIX, HIX, and P_V,n_/P_III,n_ were generally higher in amended treatments than in the control, although their responses were non-monotonic across amendment rates, suggesting greater contributions from recently produced DOM and stronger humification-related fluorescence signals. EEM-PARAFAC resolved five fluorescent components. C1, C2, C3, and C5 were predominantly humic-like, whereas C4 displayed both protein-like and humic-like features. With increasing amendment rate, the relative contributions of C1–C4 generally increased, whereas that of C5 declined, suggesting a shift from the native soil fluorescence profile toward a more complex DOM composition influenced by compost inputs and subsequent biological transformation. Chrysanthemum height, stem diameter, flower number, and biomass were generally more favorable at amendment rates of 30–40%. The entropy-weighted evaluation yielded the highest overall response score at 30%, while the 35% treatment also maintained a high score. Considering WE-DOM fluorescence characteristics, growing-medium properties, and plant performance together, a volumetric amendment rate of 30–35% represents a relatively favorable range for the tested composted material under the present pot-experiment conditions.

## 1. Introduction

Agricultural and forestry organic wastes are important organic-carbon resources generated during crop production, livestock farming, and forestry processing, including crop residues, livestock manure, spent mushroom substrate, fruit shells, branches, and leaf litter [1,2,3,4]. After composting and maturation, these materials can be applied to soil–plant systems as organic fertilizers or components of horticultural growing media, thereby supplying organic carbon, improving substrate structure, and reducing the environmental burden of waste disposal [5,6,7]. In greenhouse horticulture, the performance of compost-based amendments depends on feedstock composition, compost maturity, salinity risk, and amendment rate [8]. An insufficient amendment rate may provide limited improvement in soil quality or nutrient supply, whereas excessive incorporation can lead to unfavorable pore conditions, salt accumulation, nutrient imbalance, or phytotoxicity. Accordingly, the formulation of soil-based growing media requires an amendment range that balances substrate quality with plant growth rather than simply maximizing the proportion of composted material.

Dissolved organic matter (DOM) is one of the most mobile and reactive fractions of soil organic matter and responds rapidly to external organic inputs [9,10]. DOM provides readily available carbon for microorganisms and contains carboxyl, hydroxyl, and phenolic groups that participate in the complexation, sorption, and transport of nitrogen, phosphorus, and metals [11,12]. Compared with relatively slowly changing bulk properties such as soil organic matter and bulk density, DOM quantity and optical characteristics, including source-related, humification-related, and protein-like fluorescence features, are often more sensitive to organic inputs and biological processing [10,12,13]. During composting, organic matter undergoes decomposition, resynthesis, and humification, and the spectral characteristics of compost-derived DOM can provide information on stabilization and potential environmental behavior [14,15]. DOM therefore offers a process-sensitive complement to conventional indicators when evaluating growing-medium formulations.

Excitation-emission matrix fluorescence spectroscopy (EEM) is widely used to characterize DOM in soils, composts, and aquatic systems because it requires only a small sample volume, is highly sensitive, and rapidly resolves fluorescent fractions [12]. When combined with parallel factor analysis (PARAFAC), EEM data can be decomposed into relatively independent fluorescent components, allowing treatment-dependent changes in protein-like, fulvic-like, and humic-like signals to be compared [12,16]. The fluorescence index (FI), biological index (BIX), and humification index (HIX) are commonly used as optical indicators of source-related characteristics, recently produced autochthonous contributions, and humification, while fluorescence regional integration (FRI) quantifies fluorescence responses across five operational EEM regions [17]. These indices and PARAFAC components describe optical properties of DOM; they should not be treated as direct measurements of microbial activity or specific biological processes. Nevertheless, EEM-PARAFAC provides complementary information on the soluble organic-matter pool that conventional nutrient and physical measurements do not directly capture [18,19]. Previous applications have focused largely on compost maturity and humification of compost-derived DOM [14,15,20,21,22]. By contrast, much less attention has been paid to the use of EEM-PARAFAC as a tool for evaluating compost-amended horticultural growing media in which substrate physicochemical quality and crop performance must be considered simultaneously. Systematic evidence remains limited on how WE-DOM fluorescence composition varies across compost amendment rates and how these changes co-vary with substrate physicochemical properties and horticultural plant performance. This gap is particularly relevant to the optimization of soil-based horticultural growing media.

Chrysanthemum is an important potted and cut-flower crop whose growth and ornamental quality are highly responsive to substrate conditions [23]. Previous studies have shown that chrysanthemum performance is sensitive to salinity, nutrient balance, aeration, and water retention, and that appropriate combinations of organic substrate components and cultivation management can improve agronomic performance and biomass accumulation [24,25,26]. However, horticultural studies have generally emphasized plant production, whereas environmental spectroscopy studies have focused on DOM characteristics; integration of these perspectives in compost-based growing-medium optimization remains limited.

This study used composted agricultural and forestry organic materials prepared from chestnut shells, chicken manure, and spent mushroom substrate as an amendment in a chrysanthemum pot experiment. Eleven volumetric amendment rates, ranging from 0% to 50%, were evaluated to characterize coordinated responses in WE-DOM fluorescence composition, growing-medium physicochemical properties, and plant growth, and to identify a relatively suitable amendment range using entropy-weighted comprehensive evaluation. The study addressed three questions: (1) how amendment rate affects WE-DOM source-related and humification-related optical characteristics; (2) how WE-DOM fluorescent components are associated with nutrient accumulation and physical properties of the growing medium; and (3) which amendment range provides the most balanced response across WE-DOM characteristics, substrate quality, and chrysanthemum growth. We hypothesized that increasing compost amendment would systematically alter WE-DOM fluorescence composition and humification-related characteristics; that an appropriate amendment rate would be associated with improved substrate properties and chrysanthemum growth; and that excessively high amendment rates would weaken these favorable plant responses.

## 2. Results

### 2.1. Compost Amendment Alters WE-DOM Source-Related and Humification Indices

**Figure 1:**
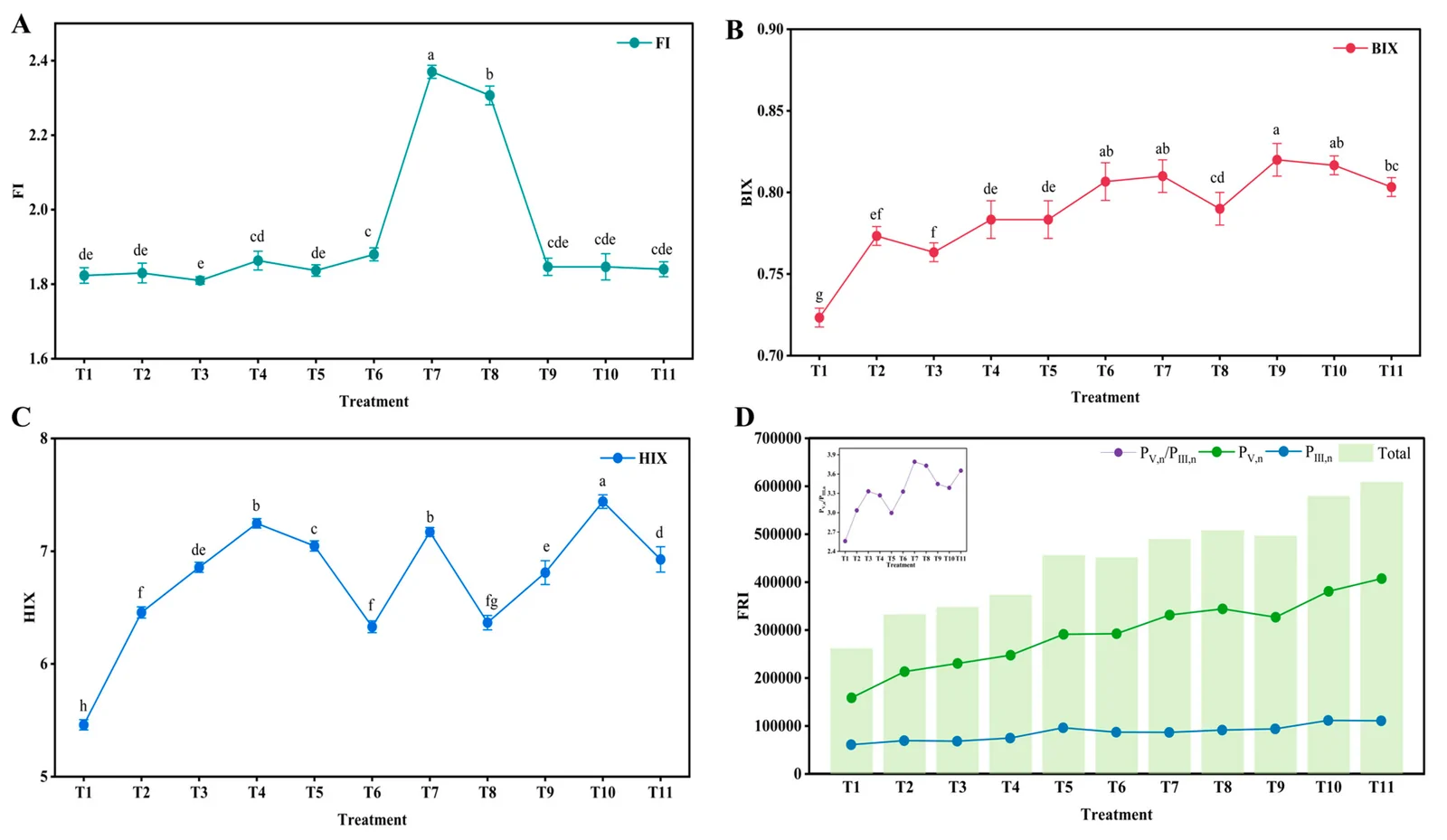
WE-DOM Fluorescence Parameters and Fluorescence Regional Integration Characteristics under Different Compost Amendment Rates. Note: T1-T11 represent 0–50% (*v*/*v*) compost amendment, respectively. (**A**) fluorescence index (FI); (**B**) biological index (BIX); (**C**) humification index (HIX); (**D**) fluorescence regional integration (FRI). P_III,n_ and P_V,n_ denote the normalized integration values of the fulvic-like and humic-like regions, respectively; P_V,n_/P_III,n_ describes the humic-like fluorescence signal relative to the fulvic-like signal. Different lowercase letters indicate significant differences among treatments for the same indicator (*p* < 0.05).

FI, BIX, and HIX differed markedly among amendment treatments (Table S1, Figure 1). FI ranged from 1.810 to 2.371, with the highest value in T7 (2.371) followed by T8 (2.308); both exceeded 1.9. These values indicate that WE-DOM at amendment rates of 30–35% exhibited a stronger microbial-source-related optical signature. Higher FI is often associated with greater microbial-source contributions or lower aromaticity, although in a soil-compost-plant system the observed signal may also reflect contributions from the compost itself, root-derived inputs, and microbial processing [11,14]. FI values in the remaining treatments were generally between 1.82 and 1.95, consistent with mixed source characteristics.

BIX was generally higher in amended treatments, although its response was not strictly monotonic with the amendment rate. T9 and T10 had relatively high values of 0.819 and 0.815, respectively, indicating a greater relative contribution of recently produced autochthonous fluorescence at medium-to-high amendment rates. HIX ranged from 5.460 to 7.440. The control (T1) had a value of 5.460, whereas most amended treatments had higher HIX values, with the maximum in T10 (7.440). Taken together, FI, BIX, and HIX indicate that the 30–35% treatments combined relatively strong microbial-source-related optical characteristics with pronounced humification-related fluorescence, while higher amendment rates tended to maintain stronger humification signals.

FRI showed that, after normalization to the same DOC concentration, the total fluorescence response generally increased with compost amendment rate. Total FRI increased from 262,247.0 in T1 to 609,102.1 in T11, an increase of 132.3%, indicating a greater response of fluorescence-active fractions per unit DOC in amended soils.

The relative contribution of Peak III (fulvic-like fluorescence) declined from 23.21% in T1 to 17.68% in T7, whereas P_V,n_/P_III,n_ increased from 2.54 to 3.79, representing a 48.4% increase. Thus, at the 30% amendment rate, humic-like fluorescence was enhanced relative to fulvic-like fluorescence. Although total FRI continued to increase at higher amendment rates, P_V,n_/P_III,n_ did not increase monotonically and remained relatively high around 30–35%. This divergence indicates that greater WE-DOM fluorescence intensity and stronger humification-related optical characteristics did not develop in parallel and that intermediate amendment rates produced a more balanced fluorescence profile.

### 2.2. Characteristics of WE-DOM Fluorescent Components Resolved by EEM-PARAFAC

**Figure 2:**
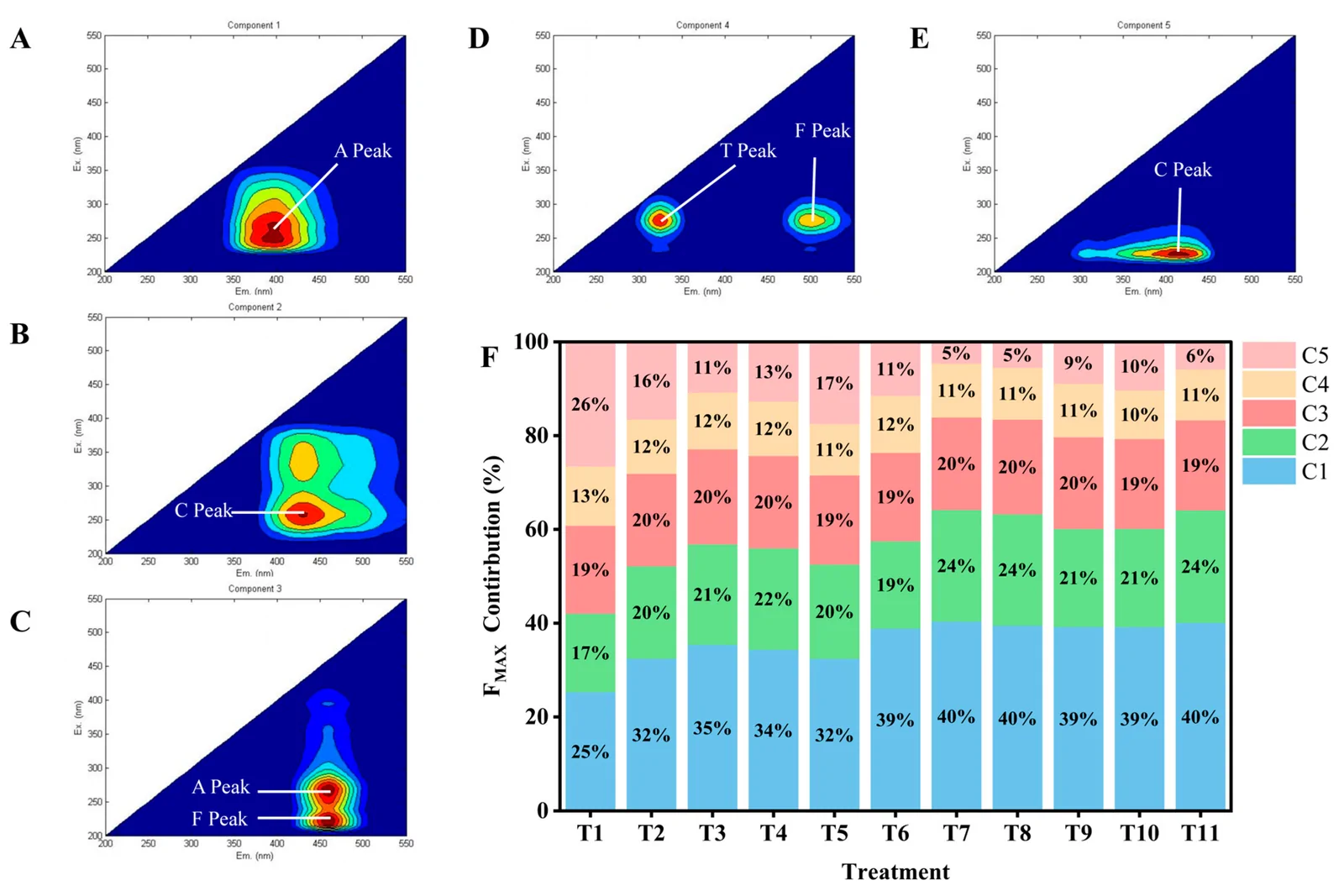
Spectral Profiles of WE-DOM Fluorescent Components and Relative Fmax Contributions under Different Compost Amendment Rates. Note: (**A**–**E**) represent the five fluorescent components (C1–C5) resolved by EEM-PARAFAC; (**F**) shows the relative contribution of Fmax for each component under different amendment rates.

The PARAFAC model resolved five fluorescent components (Table 1, Figure 2). Component identities are presented as tentative spectral assignments based on excitation/emission maxima and comparison with similar PARAFAC components reported in the literature, rather than as definitive chemical identifications. C1 showed a characteristic peak at approximately Ex/Em = 250/395 nm and was assigned as a UV fulvic-like component. C2 peaked at approximately 260/425 nm and was assigned as humic-like; its red-shifted spectrum relative to C1 is consistent with greater aromaticity and structural complexity. C3 exhibited two excitation maxima at approximately 225 and 265 nm with an emission maximum near 460 nm and was assigned as fulvic-/humic-like. C4 showed fluorescence features near 275/325 and 275/500 nm, consistent with mixed tryptophan-like protein and humic-like characteristics. C5 peaked near 225/415 nm and was assigned as a UV humic-like component. Overall, humic-like fluorescence dominated the WE-DOM spectra, whereas protein-like fluorescence was comparatively weak but responsive to amendment.

The relative proportions of the maximum fluorescence intensity (Fmax) of different PARAFAC components varied significantly among treatments with different compost amendment rates. In the T1 treatment without organic material addition, C5 exhibited the highest relative contribution, accounting for approximately 25.9%, indicating that soil WE-DOM was primarily controlled by background humic-like fluorescence signals. After the addition of organic materials, C1 became the dominant component in all amended treatments, with relative contributions ranging from approximately 31.4% to 38.4%. The relative contributions of C1, C2, C3, and C4 generally increased, whereas the contribution of C5 decreased. These results suggested that organic material inputs shifted the fluorescent composition of soil WE-DOM from being dominated by native soil background humic-like signals toward a more complex fluorescence profile consistent with contributions from exogenous organic matter and biological processing. It should be noted that PARAFAC components represent statistically derived fluorescence response patterns and cannot be directly equated with specific chemical compounds. Therefore, the observed changes should not be interpreted as a unidirectional transformation of one WE-DOM component into another.

### 2.3. Compost Amendment Alters Growing-Medium Chemical and Physical Properties

Compost amendment significantly altered the chemical properties of the growing medium (Table S2). SOM increased from 31.87 g kg^−1^ in T1 to 109.55 g kg^−1^ in T11, a 243.7% increase, while DOC increased from 0.23 to 0.72 g kg^−1^, a 213.0% increase. TN and TP also increased overall with the amendment rate; in T11, TN and TP reached 3.28 and 3.53 g kg^−1^, respectively, representing increases of 118.7% and 88.8% relative to T1. Most amended treatments had lower pH than the control, and pH declined to 6.36 in T11, indicating a shift from slightly alkaline toward neutral or mildly acidic conditions. Germination index (GI) was higher than in T1 for most amended treatments and reached 118.94% in T10, indicating no evident phytotoxic response within the amendment range tested.

Compost amendment also altered the physical properties of the growing medium (Table S3). Relative to T1, most amended treatments had lower bulk density (BD) and higher soil water content (SWC), field capacity (FC), total porosity (Pt), and capillary porosity (CP). BD ranged from 0.76 to 1.47 g cm^−3^. FC and Pt were generally higher at medium-to-high amendment rates; for example, SWC in T9 and T10 was 23.39% and 23.32%, FC was 59.48% and 61.49%, and Pt was 61.32% and 60.60%, respectively. These measured changes show that compost amendment was associated with lower bulk density and improved water-retention-related and overall porosity characteristics; they are not interpreted as direct evidence of improved aeration because air-filled porosity and gas diffusion were not measured.

### 2.4. Effects of Compost Amendment on Chrysanthemum Growth

**Figure 3:**
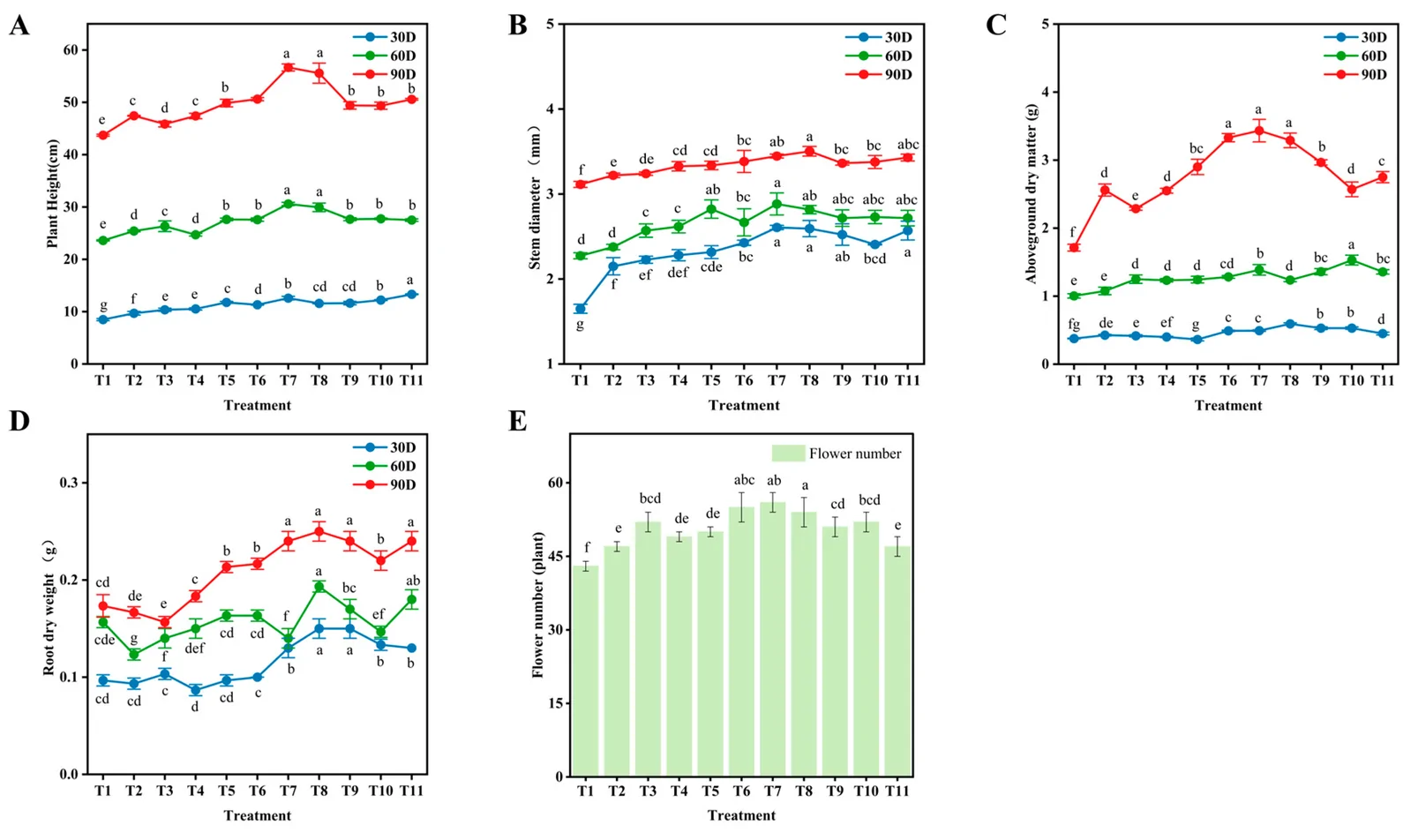
Effects of Compost Amendment Rate on Chrysanthemum Agronomic Traits. Note: (**A**) plant height; (**B**) stem diameter; (**C**) aboveground dry matter; (**D**) belowground dry matter; (**E**) flower number. Different lowercase letters indicate significant differences among treatments within the same growth stage or for the same indicator (*p* < 0.05).

Compost amendment significantly affected chrysanthemum height, stem diameter, and flower production (Table S4, Figure 3). At flowering, plant height was greatest in T7 and T8 (56.67 and 55.57 cm), corresponding to 1.30 and 1.27 times the value in T1. Stem diameter was highest in T8 (3.50 cm), 1.12 times that in T1, while flower number reached a maximum of 56 per plant in T7, 1.30 times the control. These results indicate that intermediate amendment rates were associated with more favorable vegetative and reproductive growth, whereas further increases in amendment rate did not produce corresponding increases in plant height or flower number.

Biomass responses further supported a non-linear response to compost amendment. At flowering, T8 had the highest aboveground fresh weight (28.51 g), 2.06 times the value in T1, while T7 had the highest aboveground dry weight (3.43 g), 2.00 times the control. T8 also maintained relatively high belowground fresh and dry weights (1.69 and 0.25 g), corresponding to 1.46 and 1.47 times T1, respectively. Overall, chrysanthemum biomass was more favorable at amendment rates of 30–40%, suggesting that increasing compost rates did not produce a linear gain in plant growth and that higher amendment rates were not necessarily beneficial.

### 2.5. Coupling Relationships Between WE-DOM Fluorescence Parameters and Soil–Plant Responses

**Figure 4:**
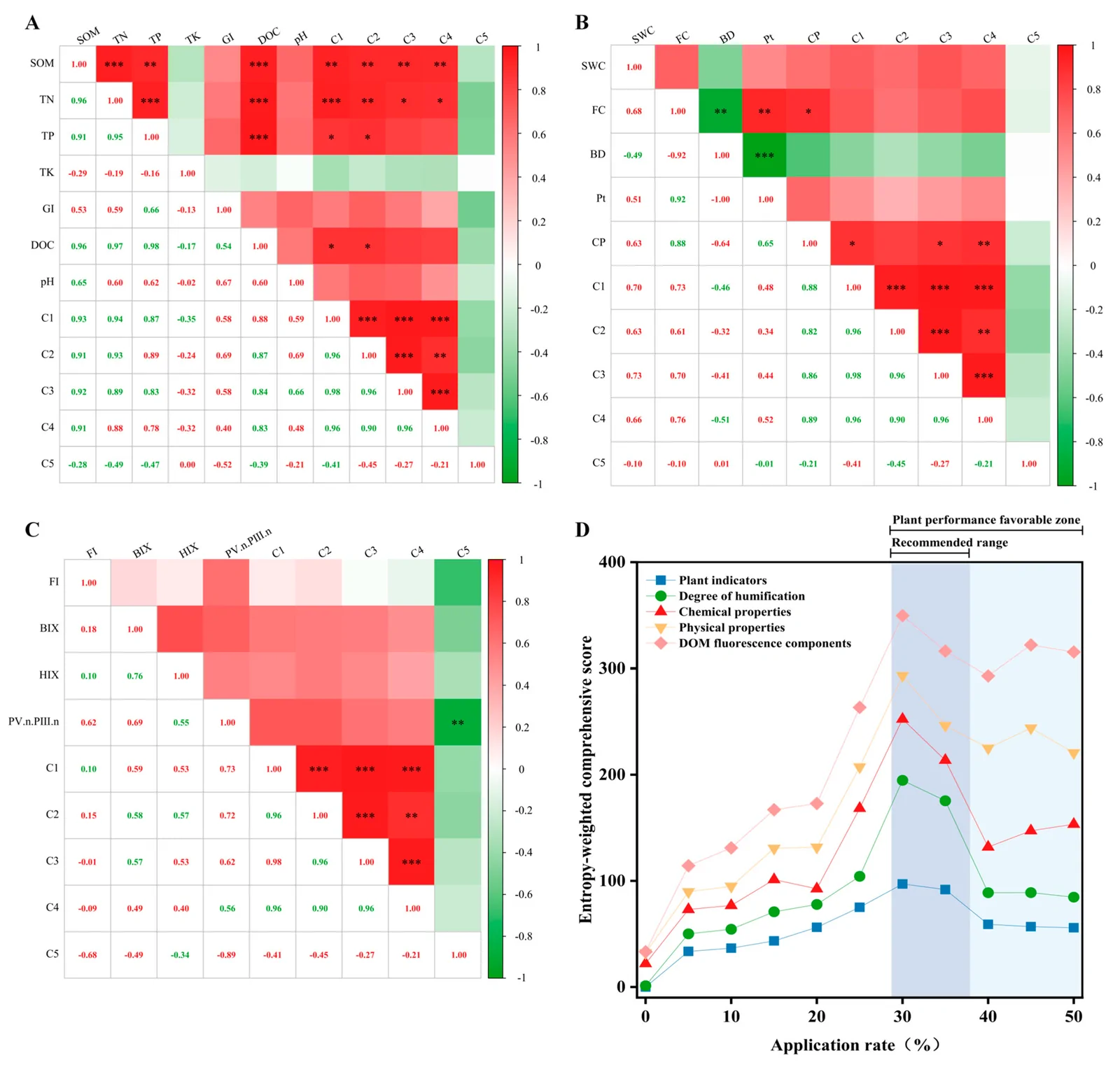
Coupling Relationships among Growing-Medium Properties, WE-DOM Fluorescent Components, and Comprehensive Response Indices. Note: (**A**) correlations between chemical properties and PARAFAC components; (**B**) correlations between physical properties and PARAFAC components; (**C**) correlations between WE-DOM fluorescence indices and PARAFAC components; (**D**) entropy-weighted scores of the five evaluation dimensions across amendment rates. D presents the final integrated scores of the five evaluation dimensions across amendment rates, rather than cumulative curves of dimension-specific scores. Treatment performance should therefore be interpreted together with the final comprehensive response score. In the heatmaps, red indicates positive correlation and green indicates negative correlation; values are Pearson correlation coefficients, and *, **, and *** indicate *p* < 0.05, *p* < 0.01, and *p* < 0.001, respectively. Dark shading denotes the relatively favorable 30–35% amendment range, whereas light shading denotes the 30–40% range in which chrysanthemum growth was relatively favorable. SOM, soil organic matter; TN, total nitrogen; TP, total phosphorus; TK, total potassium; GI, germination index; DOC, dissolved organic carbon; SWC, soil water content; FC, field capacity; BD, bulk density; Pt, total porosity; CP, capillary porosity; FI, fluorescence index; BIX, biological index; HIX, humification index; C1–C5, WE-DOM fluorescent components resolved by EEM-PARAFAC.

Significant associations were observed between WE-DOM fluorescence indices and PARAFAC components (Figure 4C). BIX was positively correlated with C1-C4 and negatively correlated with C5, while HIX was positively correlated with C1-C3. FI was negatively correlated with C5. Because FI, BIX, and HIX are empirical optical indices, these relationships are interpreted jointly with PARAFAC components and growing-medium properties rather than as direct evidence of microbial activity or humification processes.

C1–C4 were generally positively associated with SOM, TN, TP, and DOC, indicating coordinated variation between fluorescent-active WE-DOM fractions and nutrient accumulation following compost amendment. C1–C4 were also positively associated with field capacity and capillary porosity and negatively associated with bulk density. These associations show that changes in WE-DOM fluorescence composition co-varied with measured physical properties of the growing medium. By contrast, C5 was negatively associated with most nutrient and humification-related variables, suggesting that it more strongly represented the relative contribution of background soil humic-like fluorescence than newly enriched WE-DOM fractions. These correlations are exploratory associations and do not establish directionality or causation.

The entropy-weighted evaluation showed an amendment-rate-dependent multidimensional response across amendment rates. The five-dimensional comprehensive score increased from 6.6 in the control (0%) to a maximum of 69.9 at 30%. Scores subsequently declined but remained relatively high at 35%, 40%, 45%, and 50% (63.2, 58.6, 64.4, and 63.1, respectively). The 30% treatment exceeded the 35%, 45%, and 50% treatments by 10.55%, 8.55%, and 10.85%, respectively, indicating that the vicinity of 30% provided the most favorable overall balance among the evaluated dimensions, with 35% representing a closely favorable level.

Dimension-specific scores revealed contrasting response patterns. The plant performance score peaked at 97.1 at 30% and remained high at 91.9 at 35%, only 5.41% below the maximum. At 40%, 45%, and 50%, the plant score declined to 59.1, 56.9, and 55.9, respectively, corresponding to decreases of 39.16%, 41.41%, and 42.48% relative to 30%. The humification-characteristic score also peaked at 30% and remained high at 35% before declining at rates above 40%. In contrast, scores for chemical properties, physical properties, and WE-DOM fluorescent components remained relatively favorable at higher amendment rates. Thus, increased nutrient accumulation and some fluorescence responses at high amendment rates were not accompanied by higher plant performance. Overall, 30–35% provided the most balanced response among WE-DOM fluorescence characteristics, humification-related optical characteristics, growing-medium quality, and chrysanthemum growth.

## 3. Discussion

### 3.1. Compost Amendment Alters the Optical Source Characteristics and Fluorescent Component Structure of WE-DOM

Composted agricultural and forestry organic materials contain dissolved organic carbon, humic-substance precursors, and microbially available substrates, and their incorporation into soil is commonly accompanied by changes in DOM quantity and fluorescence composition [27]. During composting, organic matter undergoes decomposition, resynthesis, and humification, and DOM fluorescence has therefore been widely used to assess compost maturity and organic-matter stabilization [14,15,22]. In this study, compost amendment increased both soil DOC and total fluorescence intensity normalized to DOC, showing that external organic inputs enlarged the soluble organic-carbon pool and altered the relative contribution of fluorescent-active fractions. Higher FI and BIX values were consistent with greater contributions from microbially derived and recently produced DOM. These patterns may reflect contributions from labile compost-derived carbon, rhizosphere inputs, and microbial processing, but the present data support associations among response variables rather than a specific mechanistic pathway.

PARAFAC showed higher relative contributions of C1–C4 and a lower contribution of C5 following compost amendment. This pattern was consistent with the responses of FI, BIX, and HIX, indicating coordinated variation between WE-DOM quantity and fluorescent-component structure across amendment rates. Protein-like, fulvic-like, and humic-like fluorescence can respond differently to compost maturity, feedstock composition, and microbial processing [22,27,28,29]. Because the present experiment used an amendment-rate gradient rather than a time series, these results should be interpreted as cross-sectional differences among treatments. They do not demonstrate unidirectional conversion from one WE-DOM component to another or direct mechanistic regulation of plant growth by specific fluorescent components.

### 3.2. WE-DOM Optical Characteristics Co-Vary with Nutrient Accumulation and Physical Properties

DOM is a highly reactive fraction of soil organic matter and is closely associated with nutrient transformations and biological processes [9,30,31]. Compost amendment increased SOM, DOC, TN, and TP, indicating that the compost served as a source of carbon and nutrients. Most amended treatments also exhibited lower pH and bulk density together with higher porosity and water-retention-related variables. Similar changes following compost or organic amendment have been widely reported [8,32,33]. Because air-filled porosity, gas diffusivity, and oxygen status were not measured, however, these results are restricted to the measured physical properties and should not be used to infer improved aeration or specific changes in pore-size distribution.

Compared with bulk indicators such as SOM, TN, and BD, WE-DOM fluorescence provides a more responsive optical description of the soluble organic-matter pool. In the present study, BIX was positively associated with C1-C4 and HIX with C1–C3, suggesting that recently produced and humification-related optical signals were represented in the PARAFAC-resolved component structure. The coordinated variation in C1–C4 with SOM, TN, TP, DOC, field capacity, and capillary porosity further suggests that WE-DOM fluorescence may serve as a useful complementary characterization metric for describing differences in substrate conditions at harvest. These correlations do not establish direct causation. Moreover, EEM-PARAFAC captures only fluorescent fractions of DOM and does not comprehensively characterize polysaccharides, aliphatic compounds, or other weakly fluorescent or nonfluorescent molecules. WE-DOM fluorescence should therefore complement, rather than replace, molecular-level characterization. Future studies combining high-resolution mass spectrometry, NMR spectroscopy, microbial-community profiling, and enzyme activities would help resolve the molecular origins and functional significance of the observed fluorescent components [16,34].

### 3.3. Moderate Amendment Rates Favor Coordinated WE-DOM Characteristics and Chrysanthemum Growth

Higher compost amendment rates generally increased SOM and DOC, whereas chrysanthemum height, flower number, and biomass did not increase monotonically and tended to be greater at 30–40%. Chrysanthemum is sensitive to the water retention, aeration, and nutrient supply characteristics of growing media, and balanced substrate mixtures often perform better than media dominated by a single organic component [24,26]. Growing-medium optimization should therefore not aim solely to maximize SOM or DOC but should consider the integrated balance among nutrient supply, pore characteristics, water retention, aeration, and the availability of soluble organic substrates. More broadly, plant performance emerges from the combined effects of substrate quality, nutrient availability, and plant nutrient uptake and physiological processes rather than from any single physicochemical property [35].

Water-holding capacity differed among amendment treatments [36,37]. Because all treatments were irrigated at the same frequency, plants may not have experienced identical water availability. Consequently, differences in plant growth and substrate properties may reflect not only the amendment rate itself but also the interaction between substrate water retention and water supply. At high amendment rates, altered field capacity and pore characteristics may have changed the root-zone water-air environment and thereby affected nutrient uptake and chrysanthemum growth [38,39]. The positive responses to compost amendment should therefore be interpreted within an integrated framework of substrate physicochemical conditions, nutrient release, and plant nutrition. Future experiments should use irrigation control based on field capacity or defined moisture thresholds to improve comparability among substrate formulations.

At amendment rates of 30–35%, FI exceeded 1.9, BIX remained relatively high, and the humic-like/fulvic-like fluorescence ratio reached or approached its maximum. These optical characteristics coincided with favorable plant height, flower number, and aboveground dry matter. Intermediate amendment rates may therefore have provided a more coordinated combination of available carbon, nutrient release, substrate properties, and root-zone conditions. At higher rates, SOM and DOC continued to increase while gains in plant growth diminished. Potential explanations include salinity, mineral-N imbalance, organic-acid accumulation, or changes in the root-zone air-water environment, but these mechanisms cannot be distinguished because electrical conductivity, mineral N forms, organic acids, salt-ion composition, and gas diffusivity were not measured. Reduced plant performance at high amendment rates should therefore be treated as an important interpretive constraint rather than attributed to a single mechanism. In addition, this study primarily analyzed the physicochemical properties and WE-DOM fluorescence characteristics of the growing media under different treatments at the end of cultivation, whereas the initial mixed substrates prepared for each treatment were not synchronously characterized. Therefore, the differences observed among treatments may reflect not only changes induced during cultivation, such as organic matter decomposition and transformation, plant uptake, and microbial activity, but also, at least in part, initial differences already present among substrate mixtures with different amendment ratios at the beginning of the experiment. Future studies should further determine the baseline properties and spectral characteristics of the initial mixed substrates for each treatment to distinguish the relative contributions of initial substrate differences and cultivation-induced effects.

### 3.4. EEM-PARAFAC-Based Integrated Response Framework and Study Limitations

**Figure 5:**
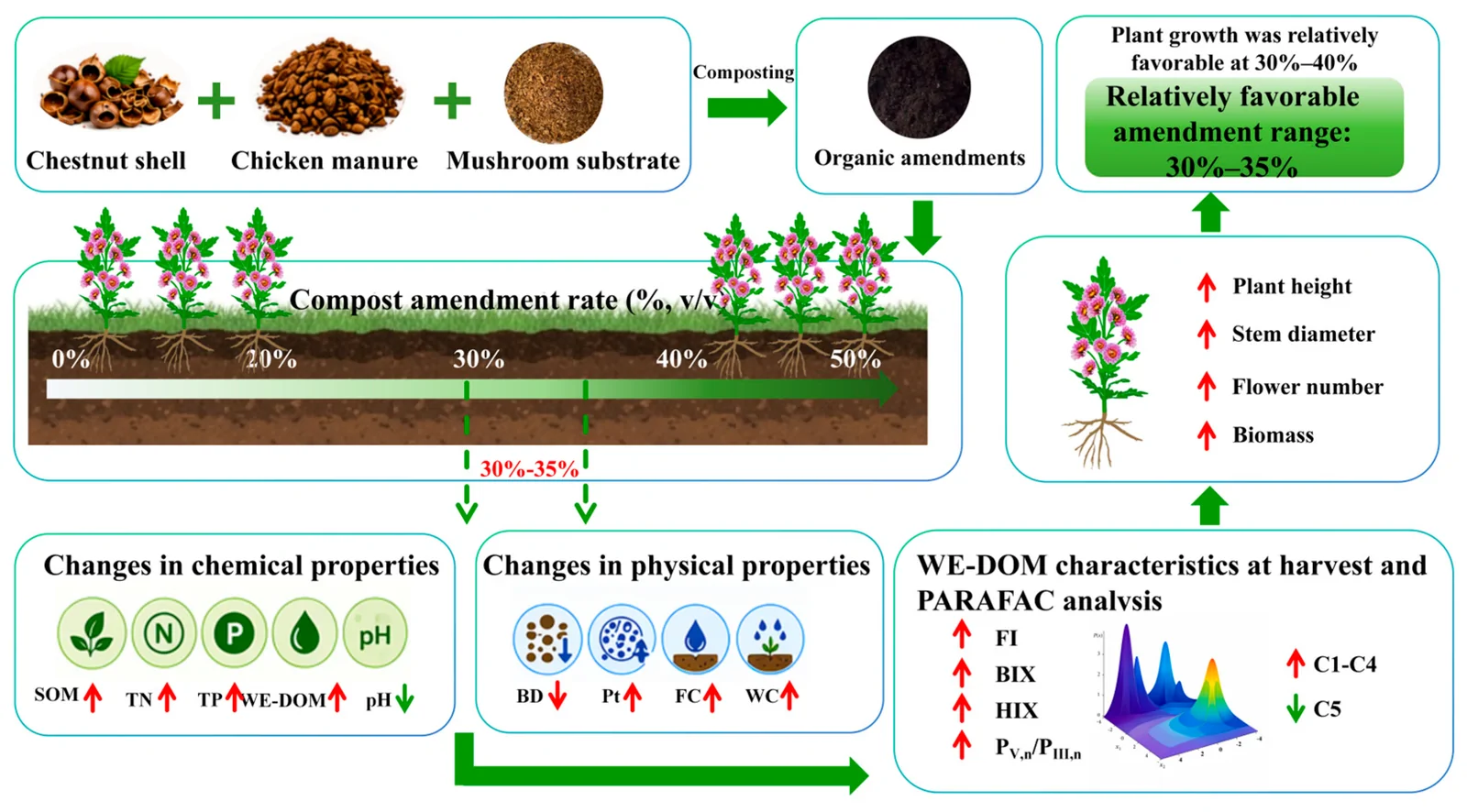
Integrated Response Framework Linking WE-DOM Characteristics at Harvest, Growing-Medium Quality, and Chrysanthemum Growth across Compost Amendment Rates. Note: The figure summarizes WE-DOM characteristics measured at harvest together with EEM-PARAFAC components, fluorescence indices, FRI, growing-medium properties, plant performance, and entropy-weighted evaluation. Arrows indicate response directions synthesized from the amendment-rate gradient and exploratory associations; they are conceptual and do not represent causal paths derived from structural equation modeling or path analysis.

Based on EEM-PARAFAC, the amendment-rate gradient, exploratory correlation analysis, and entropy-weighted evaluation, this study summarizes an integrated response framework linking WE-DOM fluorescence characteristics, growing-medium quality, and chrysanthemum performance (Figure 5). The principal contribution is the joint evaluation of PARAFAC-resolved WE-DOM components with physicochemical properties and plant responses across a 0–50% amendment gradient. The framework is based on co-variation and multidimensional evaluation rather than direct mechanistic verification. The pathways through which microbial communities transform DOM, root exudates modify DOM composition, and DOM characteristics influence nutrient availability and plant physiology remain to be established. Plant responses to organic amendments depend not only on improved substrate fertility but also on whether nutrient release is synchronized with physiological demand during growth [40]. In this study, the 30–35% treatments maintained relatively favorable WE-DOM optical characteristics, substrate quality, and plant performance, suggesting a comparatively balanced response. At higher amendment rates, some nutrient and fluorescence-component scores remained high while plant-performance scores declined, further indicating that higher substrate fertility does not necessarily translate into superior growth when nutrient supply and physiological demand are not synchronized. The entropy-weighted evaluation provides a quantitative means of integrating multiple response dimensions, but treatment ranking depends on indicator selection, the number of indicators, data dispersion, and the weighting procedure [41]. We therefore use the comprehensive score as a decision-support tool for identifying a relatively suitable amendment range rather than as the sole criterion for treatment superiority.

## 4. Materials and Methods

### 4.1. Compost Material and Experimental Soil

The composted material was produced from chestnut shells, chicken manure, and spent mushroom substrate and met the relevant requirements of the Chinese Organic Fertilizer Standard (NY/T 525-2021) after maturation [42]. Compost maturity was comprehensively assessed based on uniform appearance, a powdery or granular texture, the absence of foul odor, GI ≥ 70, OM ≥ 30%, and pH 5.5–8.5; the viable bacterial count was determined using the plate count method. The experimental soil was collected from the 0–20 cm cultivated layer of an agricultural field in Beijing, China, and classified as cinnamon soil according to the Chinese Soil Taxonomy (approximately equivalent to a Luvisol in the WRB system and an Alfisol in USDA Soil Taxonomy). The soil had a silt loam texture, comprising 27.5% sand, 50.2% silt, and 22.3% clay. Basic properties of the original composted material (after air-drying) and mineral soil before mixing are reported in Table 2.

### 4.2. Pot Experiment Design

The pot experiment was conducted in a greenhouse at the Beijing Academy of Agriculture and Forestry Sciences. Daytime temperature was maintained at 22–28 °C, nighttime temperature at 15–20 °C, and relative humidity at 50–75%. All treatments received the same natural-light exposure and greenhouse management. No additional fertilizer was applied during the experiment, so differences among treatments arose primarily from the compost amendment rate.

Chrysanthemum (*Chrysanthemum morifolium* Ramat.) cv. ‘Shenma’ plants were propagated from greenhouse cuttings. At transplanting, seedlings were uniform in vigor, approximately 10 cm tall, with a stem diameter of about 1.5 mm, and one functional leaf retained per plant. Pots had an inner diameter of 50 cm and a height of 30 cm and were filled with 58 L of mixed growing medium. The compost amendment rate was the single experimental factor, with volumetric rates of 0%, 5%, 10%, 15%, 20%, 25%, 30%, 35%, 40%, 45%, and 50%, designated T1–T11. The composted material was thoroughly mixed with the soil at the target volumetric ratio before pot filling. Each treatment comprised three independent pots, with three chrysanthemum plants per pot. The three plants within each pot were used only to obtain pot-level growth phenotypes and were not treated as independent statistical replicates. The experiment began in July and lasted 120 days. All treatments were irrigated at the same frequency.

### 4.3. Measurement of Growing-Medium Physicochemical Properties and Plant Traits

Growing-medium physicochemical properties were measured according to standard soil agrochemical methods [43,44]. pH was measured using a PHS-3C pH meter. Total nitrogen (TN) was determined by semi-micro Kjeldahl digestion; total phosphorus (TP) and total potassium (TK) were determined after microwave digestion; total organic carbon was measured with a Multi N/C 3100 analyzer (Analytik Jena AG, Jena, Germany); and soil organic matter (SOM) was determined by external-heating K_2_Cr_2_O_7_-H_2_SO_4_ oxidation. Bulk density (BD), total porosity (Pt), and capillary porosity (CP) were measured using the core method, while soil water content (SWC) and field capacity (FC) were determined using standard soil physical procedures. The germination index (GI) was determined according to the method specified in NY/T 525-2021. This index was used to evaluate the potential phytotoxicity of the substrate, with higher GI values indicating weaker inhibition of seed germination and radicle elongation.

Soil samples were collected after the plants were harvested (120 days). Undisturbed cores from the 0–20 cm layer were used for physical measurements. Composite soil samples were collected at multiple points within each pot, air-dried, and passed through a 2 mm sieve for chemical analyses; a subsample was further ground and passed through a 100-mesh sieve for WE-DOM extraction and fluorescence analysis. Accordingly, the DOM analyzed here represents water-extractable DOM obtained from air-dried soil under standardized sample preparation rather than the instantaneous composition of in situ rhizosphere DOM. Air-drying and grinding were used to standardize sample conditions across treatments and improve analytical reproducibility.

Plant traits included plant height, stem diameter, aboveground and belowground fresh and dry mass, and flower number. The experiment lasted for 120 days, from transplanting to the final destructive sampling. Plant height and stem diameter were measured on the same marked plants at 30, 60, and 90 d after transplanting. Aboveground and belowground dry mass were determined by destructive sampling at 120 d. Plant height was measured with a ruler, stem diameter with a digital caliper to 0.01 mm, and fresh and dry masses with an electronic balance to 0.01 g. Flowering was assessed at full flowering (90 d after transplanting). The pot, rather than the individual plant, was treated as the independent replicate for statistical analysis.

### 4.4. Extraction of WE-DOM and EEM Fluorescence Measurement

WE-DOM was obtained by water extraction [45]. Briefly, 5.00 g of air-dried soil was placed in a 50 mL centrifuge tube, mixed with deionized water at a 1:10 soil-to-water ratio, shaken at 250 rpm for 24 h at room temperature, and centrifuged at 10,000 rpm for 10 min. The supernatant was filtered through a 0.45 μm membrane. DOC concentration was measured with a Multi N/C 2100 TOC analyzer (Analytik Jena AG, Jena, Germany). To reduce concentration-dependent fluorescence effects, all extracts were diluted to 15 mg C L^−1^ before fluorescence measurement, stored at 4 °C in the dark, and analyzed as soon as possible. After Raman normalization, DOC fluorescence intensity was expressed in Raman units (R.U.). Total FRI primarily represented relative fluorescence response per unit DOC, whereas soil DOC concentration was determined independently by TOC analysis.

EEM spectra were measured with an F-7000 fluorescence spectrophotometer (Hitachi, Japan) using a 450 W xenon lamp and a PMT voltage of 700 V. Excitation and emission wavelengths were scanned from 200 to 600 nm at 5 nm intervals, with a scanning speed of 2400 nm min^−1^ and slit widths of 5 nm. Prior to optical analysis, the samples were further filtered through 0.2 μm membrane filters. To mitigate the inner-filter effect, samples with absorbance exceeding 0.75 at 220 nm were appropriately diluted. Absorbance spectra of the samples were measured simultaneously, and mathematical corrections based on the absorbance data were applied to eliminate the inner-filter effect. Milli-Q water was used as a blank for blank subtraction and Raman normalization, and instrument-response correction was also performed. The Rayleigh and Raman scattering regions were corrected during the preprocessing.

### 4.5. EEM-PARAFAC and Fluorescence Parameters

Fluorescence data were exported and processed using MATLAB R2014a and the DOMFluor toolbox. Rayleigh and Raman scatter regions were removed, and the EEMs were assembled into a three-way data array. All samples had been diluted to the same DOC concentration before measurement, reducing concentration-related differences among samples, although this normalization is not equivalent to an absorbance-based mathematical inner-filter correction. Model reliability and the appropriate number of components were evaluated by residual analysis and split-half validation. The dataset was randomly divided into two subsets, and PARAFAC solutions containing two to six components were fitted independently. Excitation and emission spectra were compared between split halves, with Tucker congruence coefficients >0.95 considered acceptable. The selected solution had core-consistency values >85%, randomly distributed residuals, and good spectral agreement between split halves. A five-component model was retained because it provided the most appropriate balance among residual structure, component stability, and spectral plausibility. Component assignments were based on excitation/emission maxima and published spectral analogs and are considered tentative [16].

Fluorescence indices and FRI were calculated using Origin 2019b. FI was calculated as the ratio of fluorescence intensity at Em = 450 nm to that at Em = 500 nm under Ex = 370 nm and used as an optical indicator of microbial versus terrestrial source characteristics [46]. BIX was calculated as the ratio of the fluorescence intensity at Em = 380 nm to the maximum fluorescence intensity within Em = 420–435 nm at Ex = 310 nm and was used to indicate the contribution of recently produced autochthonous organic matter. [47]. HIX was calculated from the ratio of integrated fluorescence intensity at Em = 435–480 nm to that at Em = 300–345 nm under Ex = 254 nm and used to characterize humification-related optical properties [48]. FRI was calculated across five operational EEM regions (Table 3), and P_V,n_/P_III,n_ was used to describe humic-like fluorescence relative to fulvic-like fluorescence [21].

### 4.6. Statistical Analysis and Comprehensive Evaluation

All statistical analyses were performed in SPSS 26.0. Data were first summarized at the pot level, and each pot was treated as an independent experimental replicate. Normality and homogeneity of variance were examined before inferential testing; normality was assessed using the Shapiro–Wilk test and homogeneity of variance using Levene’s test. When assumptions were not met, appropriate data transformation or non-parametric procedures were applied as needed. One-way ANOVA was used to compare growing-medium properties, final plant biomass, flower number, and other endpoint variables among compost amendment rates. When treatment effects were significant, Duncan’s multiple-range test was used for mean separation at *p* < 0.05. Plant height and stem diameter measured repeatedly at 30, 60, and 90 d were analyzed using repeated-measures ANOVA to test the effects of time, amendment rate, and their interaction. For repeated-measures ANOVA, sphericity was assessed using Mauchly’s test, and when the sphericity assumption was violated, the Greenhouse–Geisser correction was applied. Each pot was treated as an independent experimental replicate. Correlation analyses were conducted at the pot level and are presented as exploratory.

Pearson correlation analysis was used as an exploratory analysis to assess associations among WE-DOM fluorescence parameters, PARAFAC components, growing-medium properties, and plant traits. All correlation analyses were performed using the original pot-level observations (n = 33, i.e., 11 treatments × 3 replicate pots). Coefficients of variation among replicate measurements were examined to assess experimental consistency and were within reasonable ranges for the variables analyzed.

For multidimensional evaluation, treatment means were grouped into five first-level dimensions: plant growth (plant height, stem diameter, aboveground dry matter, and flower number), humification-related optical characteristics (FI, BIX, HIX, and P_V,n_/P_III,n_), chemical properties (SOM, TN, TP, DOC, and pH), physical properties (SWC, FC, and CP), and PARAFAC fluorescence components (C1–C5). A two-level entropy-weight method [41] was applied independently within each dimension. Positive indicators were normalized by min–max scaling, whereas pH was treated as a negative indicator; all remaining indicators were treated as positive indicators:

Positive indicators were normalized as

$$z_{ij}=\frac{x_{ij}-min(x_{j})}{max(x_{j})-min(x_{j})}$$

Whereas negative indicators were normalized as

$$z_{ij}=\frac{max(x_{j})-x_{ij}}{max(x_{j})-min(x_{j})}$$

where *x_ij_* is the original value of indicator j for treatment i and *z_ij_* is the normalized value. After normalization, the proportional contribution of each treatment to each indicator was calculated, followed by information entropy (*e_j_*), the divergence coefficient (*d_j_* = 1 − *e_j_*), and the entropy weight (*w_j_*).

$$p_{ij}=\frac{z_{ij}}{\sum_{i=1}^{n}z_{ij}}$$

$$e_{j}=-\frac{1}{ln\;n}\sum_{i=1}^{n}p_{ij}lnp_{ij}\;\;$$

The entropy weight of each indicator was determined from its divergence coefficient, so indicators with greater dispersion among treatments received greater objective weight.

$$w_{j}=\frac{1-e_{j}}{\sum_{j=1}^{n}\left(1-e_{j}\right)}\;\;$$

The independent score for each first-level evaluation dimension was calculated as the weighted sum of its normalized second-level indicators.

$$S_{i}=100\sum_{j=1}^{n}w_{j}z_{ij}\;\;$$

The final comprehensive response score was obtained by directly summing the five first-level dimension scores, without further normalization.

$$C_{i}=\sum_{i=1}^{5}S_{i}$$

All normalized values, indicator directions, entropy values, divergence coefficients, weights, dimension scores, and final comprehensive scores are provided in the Supplementary Materials to ensure transparency and reproducibility of the calculation.

## 5. Conclusions

This study evaluated a 0–50% volumetric gradient of composted agricultural and forestry organic materials in a cinnamon-soil-based chrysanthemum growing medium. The compost was prepared from chestnut shells, chicken manure, and spent mushroom substrate. Compost amendment increased SOM, DOC, TN, and TP and was associated with lower bulk density, greater porosity, and better water-retention properties. FI, BIX, HIX, and P_V,n_/P_III,n_ indicated systematic changes in the source-related and humification-related optical characteristics of WE-DOM, while EEM-PARAFAC resolved five fluorescent components whose relative contributions changed across amendment rates. These fluorescence responses represent optical associations and should not be interpreted as direct evidence of microbial mechanisms or unidirectional chemical transformation. Chrysanthemum height, stem diameter, flower number, and biomass showed a non-monotonic response and were generally greater at 30–40%. The entropy-weighted evaluation gave the highest comprehensive score at 30%, with the 35% treatment also showing a relatively high score. The comprehensive score depends on normalization, indicator composition, and weighting, and treatment differences were not uniform across individual traits. Therefore, the 30% and 35% ranges are interpreted as relatively favorable amendment ranges rather than unique optima. This recommendation is restricted to the compost feedstocks, soil type, chrysanthemum cultivar, irrigation regime, and pot conditions evaluated here. Integrating EEM-PARAFAC with conventional growing-medium properties and plant measurements provides a complementary framework for evaluating compost-based horticultural growing media and the valorization of agricultural and forestry organic wastes.

## Figures and Tables

**Table 1:** Fluorescent components of soil WE-DOM resolved by PARAFAC and their tentative assignments.

Component	Component Classification	Main Ex/Em Peak Position (nm)	Assigned Fluorescence Peak
C1	UV fulvic-like substance	250/395	Peak A
C2	UV humic-like substance	260/425	Peak C
C3	Fulvic-like/humic-like substance	225/460, 265/460	Peak A/Peak F
C4	Tryptophan-like/protein-like and humic-like substance	275/325, 275/500	Peak T/Peak F
C5	UV humic-like substance	225/415	Peak C

**Table 2:** Basic chemical properties of the composted organic material and experimental soil.

	Organic Matter (OM) %	TN %	TP %	TK %	pH	Viable Bacterial Count CFU g^−1^
Composted agricultural and forestry organic materials	44.98	0.22	0.93	0.54	7.31	7.41 × 10^8^
Soil	2.73	0.16	0.19	2.26	7.51	-

Note: OM, organic matter; TN, total nitrogen; TP, total phosphorus; TK, total potassium. ‘-‘ indicates not determined. Values refer to the original materials before preparation of the amended growing media.

**Table 3:** Characteristics of the five operational EEM fluorescence regions used for WE-DOM characterization.

Fluorescence Region	Major Fluorophore	Ex nm	Em nm
Peak I	Tyrosine-like protein	200–250	280–330
Peak II	Aromatic protein-like substances	200–250	330–380
Peak III	Fulvic-like substances	200–250	380–550
Peak IV	Tryptophan-like microbial metabolites	250–400	280–380
Peak V	Humic-like substances (high-molecular-weight humic acid)	250–400	380–550

## Data Availability

The original contributions presented in this study are included in the article/Supplementary Materials. Further inquiries can be directed to the corresponding author.

## Supplementary Materials

The following supporting information can be downloaded at: https://www.mdpi.com/article/10.3390/plants15162541/s1. Table S1, WE-DOM fluorescence parameters and FRI characteristics under different compost amendment rates; Table S2, Growing-medium chemical properties; Table S3, Growing-medium physical properties; Table S4, Major chrysanthemum growth and biomass indicators at flowering; and Table S5, Entropy values, divergence coefficients and entropy weights in the entropy-weighted evaluation.
