# Supplementary material for: Composted Agricultural and Forestry Organic Materials Amendment Rates Alter the Fluorescence Characteristics of WE-DOM and Chrysanthemum Growth in a Soil-Based Growing Medium
Source: Plants (Basel). 2026 Aug 21;15(16):2541. doi: 10.3390/plants15162541 (PMC13516165; doi:10.3390/plants15162541)
Supplement: Supplementary file 1 [file plants-15-02541-s001.zip › plants-4476655-supplementary.pdf]

Table S1 WE-DOM fluorescence parameters and FRI characteristics under different compost amendment rates

Treatment	Addition ratio (%)	FI	BIX	HIX	FRI	$P_{III,n}$ (%)	$P_{V,n}$ (%)	$P_{V,n}/P_{III,n}$
T1	0	1.823 ± 0.005d	0.724±0.012e	5.460 ± 0.556b	262246.9±331.1k	23.2±0.3a	59.4±0.0g	2.6±0.03j
T2	5	1.833 ± 0.012cd	0.772 ± 0.001cd	6.458 ± 0.150ab	332982.4±255.6j	20.8±0.2c	63.2±0.1f	3.0±0.02h
T3	10	1.810 ± 0.032d	0.763 ± 0.015d	6.859 ± 0.398ab	348163.7±790.5i	19.6±0.0e	65.3±0.0cd	3.3±0.00f
T4	15	1.863 ± 0.033cd	0.783 ± 0.002cd	7.247 ± 0.723ab	373750.0±544.0h	20.0±0.0d	65.4±0.1c	3.3±0.00g
T5	20	1.836 ± 0.001cd	0.783 ± 0.003cd	7.047 ± 0.564ab	456415.4±74.3f	21.1±0.2b	63.2±0.2f	3.0±0.02i
T6	25	1.882 ± 0.001c	0.804 ± 0.004ab	6.331 ± 0.929ab	451663.5±785h	19.3±0.1f	64.1±0.1e	3.3±0.02f
T7	30	2.371 ± 0.006a	0.808 ± 0.002ab	7.172 ± 0.079ab	489869.5±475.2e	17.7±0.0i	67.1±0.0a	3.8±0.00a
T8	35	2.308 ± 0.013b	0.791 ± 0.002bc	6.366 ± 0.957ab	508155.3±441.2c	18.0±0.1h	67.1±0.1a	3.7±0.02b
T9	40	1.845 ± 0.010cd	0.819 ± 0.001a	6.809 ± 0.112ab	496570.4±974.3d	18.9±0.1g	65.2±0.1d	3.4±0.01d
T10	45	1.845 ± 0.021cd	0.815 ± 0.003a	7.440 ± 0.151a	579874.4±972.1b	19.2±0.0f	65.2±0.0d	3.4±0.00e
T11	50	1.841 ± 0.005cd	0.803 ± 0.001ab	6.926 ± 0.428ab	609102.1±876.6a	18.2±0.1h	66.4±0.1b	3.7±0.03c

Notes: Different lowercase letters within the same column indicate significant differences among treatments ($P < 0.05$).

Table S2 Growing-medium chemical properties

Treatment	Addition ratio (%)	SOM ($g·kg^{-1}$)	TN ($g·kg^{-1}$)	TP ($g·kg^{-1}$)	TK ($g·kg^{-1}$)	GI (%)	DOC ($g·kg^{-1}$)	pH
T1	0	31.87±0.39k	1.50±0.16c	1.87±0.14d	16.34±0.13a	76.29±0.08i	0.23±0.010i	7.57±0.14a
T2	5	40.94±0.52j	1.50±0.10c	2.44±0.24b	16.35±0.08a	100.95±0.61d	0.30±0.004h	7.17±0.13ab
T3	10	44.45±0.13i	1.69±0.23c	2.39±0.18b	16.50±0.26a	97.69±0.26e	0.33±0.009g	7.11±0.16ab
T4	15	50.42±0.21h	1.68±0.20c	2.39±0.15b	16.23±0.18a	88.46±0.24f	0.35±0.005f	7.06±0.24ab
T5	20	64.75±0.06e	2.44±0.28b	2.69±0.15bc	16.64±0.08a	86.80±0.36g	0.45±0.011e	6.95±0.13bc
T6	25	64.34±0.26f	2.52±0.18b	2.31±0.19cd	15.53±0.11b	78.60±0.21h	0.45±0.007e	6.42±0.19cd
T7	30	76.40±0.07d	2.86±0.10ab	3.40±0.25a	16.25±0.09a	102.16±1.03d	0.61±0.007c	6.92±0.14bc
T8	35	60.48±0.05g	2.34±0.12b	2.73±0.15bc	15.42±0.17b	110.35±0.11c	0.37±0.008f	6.90±0.20bc
T9	40	85.44±0.15c	2.70±0.04b	2.71±0.04bc	16.63±0.07a	86.59±0.16g	0.52±0.008d	7.19±0.14ab
T10	45	103.77±0.09b	2.64±0.27b	3.13±0.09ab	15.59±0.09b	118.94±0.37a	0.65±0.006b	6.77±0.17bcd
T11	50	109.55±0.18a	3.28±0.12a	3.53±0.09a	16.29±0.12a	116.77±0.48b	0.72±0.012a	6.36±0.17d

Notes: Different lowercase letters within the same column indicate significant differences among treatments ($P < 0.05$). Abbreviations: SOM, soil organic matter; TN, total nitrogen; TP, total phosphorus; TK, total potassium; GI, germination index; DOC, dissolved organic carbon.

Table S3 Growing-medium physical properties

Treatment	Addition ratio/%	SWC/%	FC/%	BD/g·cm^{-3}	Pt/%	CP/%
T1	0	12.43±0.22h	41.62±0.16i	1.25±0.028c	53.83±0.09f	41.04±0.78g
T2	5	16.46±0.19c	33.43±0.18k	1.47±0.019a	45.39±0.07j	33.14±0.04i
T3	10	12.32±0.10h	42.21±0.11h	1.27±0.025ab	51.16±0.09g	41.48±1.25g
T4	15	14.53±0.18f	48.39±0.18e	0.76±0.320c	58.57±0.28c	48.06±0.38e
T5	20	15.36±0.09d	44.79±0.07g	1.31±0.015ab	50.53±0.21h	44.12±0.81f
T6	25	13.66±0.11g	51.46±0.27d	1.14±0.044ab	58.39±0.16c	51.04±0.5d
T7	30	14.73±0.09f	47.70±0.10f	1.22±0.015ab	53.41±0.10f	47.22±0.46e
T8	35	16.60±0.13c	39.58±0.07j	1.33±0.019ab	49.60±0.27i	39.13±1.51h
T9	40	23.39±0.28a	59.48±0.22b	1.07±0.033b	61.32±0.10a	58.37±0.94b
T10	45	23.32±0.14a	61.49±0.23a	1.07±0.021b	60.60±0.29b	60.11±0.39a
T11	50	17.55±0.19b	54.44±0.17c	1.13±0.027b	55.59±0.19d	54.13±1.59c

Notes: Abbreviations: SWC, soil water content; FC, field capacity; BD, bulk density; Pt, total porosity; CP, capillary porosity. Different lowercase letters within the same column indicate significant differences among treatments ($P < 0.05$).

Table S4 Major chrysanthemum growth and biomass indicators at flowering

Treatment	Addition ratio /%	Plant height /cm	Stem diameter /mm	Flower number /plant	Aboveground dry number/g	Root dry matter /g
T1	0	43.7±0.2e	3.1±0.0f	43±1f	1.7±0.1f	0.17±0.01cd
T2	5	47.4±0.1c	3.2±0.0e	47±1e	2.6±0.1d	0.17±0.01de
T3	10	45.9±0.5d	3.2±0.0de	52±2bcd	2.3±0.0e	0.16±0.01e
T4	15	47.4±0.5c	3.2±0.1cd	49±1de	2.6±0.0d	0.18±0.01c
T5	20	49.9±0.7b	3.3±0.1cd	50±1de	2.9±0.1bc	0.21±0.01b
T6	25	50.6±0.3b	3.4±0.1bc	55±3abc	3.3±0.1a	0.22±0.01b
T7	30	56.7±0.7a	3.4±0.0ab	56±2ab	3.4±0.2a	0.24±0.01a
T8	35	55.6±1.9a	3.5±0.1a	54±3a	3.3±0.1a	0.25±0.01a
T9	40	49.4±0.7b	3.4±0.0bc	51±2cd	3.0±0.0b	0.24±0.01a
T10	45	49.4±0.7b	3.4±0.1bc	52±2bcd	2.6±0.1d	0.22±0.01b
T11	50	50.6±0.2b	3.4±0.0abc	47±2e	2.8±0.1c	0.24±0.01a

Notes: Different lowercase letters within the same column indicate significant differences among treatments ($P < 0.05$).

Table S5 Entropy values, divergence coefficients and entropy weights in the entropy-weighted evaluation.

Dimension	Indicator	Entropy	Difference	Weight
Plant	Height	0.91	0.09	0.32
Plant	Stem	0.94	0.06	0.23
Plant	Dry Matter	0.94	0.06	0.22
Plant	Flowers	0.93	0.07	0.24
Humification	FI	0.62	0.38	0.69
Humification	BIX	0.94	0.06	0.10
Humification	HIX	0.95	0.05	0.10
Humification	$P_{V,n}$_$P_{III,n}$	0.94	0.06	0.11
Chemical	SOM	0.86	0.14	0.24
Chemical	TN	0.91	0.09	0.16
Chemical	TP	0.92	0.08	0.13
Chemical	DOC	0.92	0.08	0.15
Chemical	pH	0.82	0.18	0.31
Physical	SWC	0.83	0.17	0.48
Physical	FC	0.91	0.09	0.24
Physical	CP	0.90	0.10	0.28
PARAFAC	C1	0.92	0.08	0.17
PARAFAC	C2	0.92	0.08	0.18
PARAFAC	C3	0.92	0.08	0.18
PARAFAC	C4	0.90	0.10	0.24
PARAFAC	C5	0.90	0.10	0.23
